# Cerebral Arterial Air Embolism after Diagnostic Flexible Fiberoptic Bronchoscopy: A Case Report and Review of the Literature

**DOI:** 10.1155/2018/7108215

**Published:** 2018-05-07

**Authors:** Keita Maemura, Hidenori Kage, Hideaki Isago, Hideyuki Takeshima, Kosuke Makita, Yosuke Amano, Daiya Takai, Nobuya Ohishi, Takahide Nagase

**Affiliations:** ^1^Department of Respiratory Medicine, The University of Tokyo Graduate School of Medicine, Tokyo, Japan; ^2^Department of Clinical Laboratory, The University of Tokyo Hospital, Tokyo, Japan

## Abstract

Cerebral arterial air embolism (CAAE) is an extremely rare complication of diagnostic flexible fiberoptic bronchoscopy, reported to occur once about every 103978 examinations. In all the eight cases of CAAE reported previously, the patients had undergone transbronchial lung biopsy (TBLB) or transbronchial needle aspiration (TBNA) prior to the onset of CAAE. Herein, we describe the case of a 77-year-old patient with double primary lung cancer who developed CAAE after bronchial curette cytology, which is considered to be less invasive than TBLB or TBNA. The patient was treated with supplemental oxygen, but paresis of the left upper arm and left spatial neglect remained. This is the first report of CAAE occurring after bronchial curettage during diagnostic flexible fiberoptic bronchoscopy.

## 1. Introduction

Arterial air embolism (AAE) is known to occur after scuba diving or as an iatrogenic complication. It is a well-known complication of CT-guided lung biopsy but occurs much less frequently as a complication of diagnostic flexible fiberoptic bronchoscopy. According to a nationwide survey of the complications of bronchoscopy conducted in 2010 in Japan, in which questionnaires were collected from 483 out of 538 facilities certificated by the Japan Society for Respiratory Endoscopy, AAE was reported at a frequency of about once every 103978 examinations (0.00096%) [1]. This frequency is much lower than that reported in patients undergoing CT-guided lung biopsy, in whom AAE is known to occur at a frequency of 0.06 to 0.45% [2, 3]. Of all types of AAE, coronary AAE and cerebral AAE are the most serious and potentially fatal. Herein, we present a patient who developed cerebral arterial air embolism (CAAE) after diagnostic flexible fiberoptic bronchoscopy.

## 2. Case Presentation

**Figure 1:**
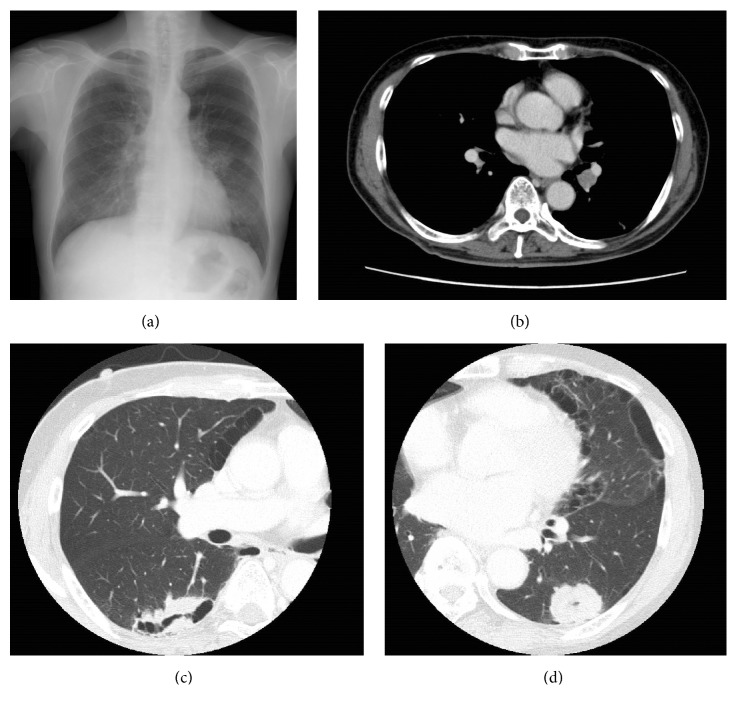
Radiologic examinations at the first visit to our hospital. (a) Chest X-ray. (b–d) Contrast-enhanced CT scan. (b) The left hilar lymph node. (c) The mass in the right lung. (d) The mass in the left lung.

A 77-year-old male patient with a smoking history of 55 pack-years was referred to our hospital with an abnormality on the chest X-ray detected during his annual medical checkup (Figure 1).

Contrast-enhanced CT revealed a cavitary mass measuring 50 mm in diameter in the right lower lobe, a mass measuring 34 mm in diameter in the left lower lobe, and an enlarged left hilar lymph node (#11) (Figure 1). No metastatic lesions were detected. Based on these findings, the patient was strongly suspected as having a double primary lung cancer.

At the first admission, flexible fiberoptic bronchoscopy was performed, and the cytological findings in endobronchial ultrasound-guided transbronchial needle aspiration (EBUS-TBNA) specimens obtained from the left hilar lymph node were classified as class V, squamous cell carcinoma. After the examination, slight left pneumothorax was noted, which improved spontaneously without additional treatment.

**Figure 2:**
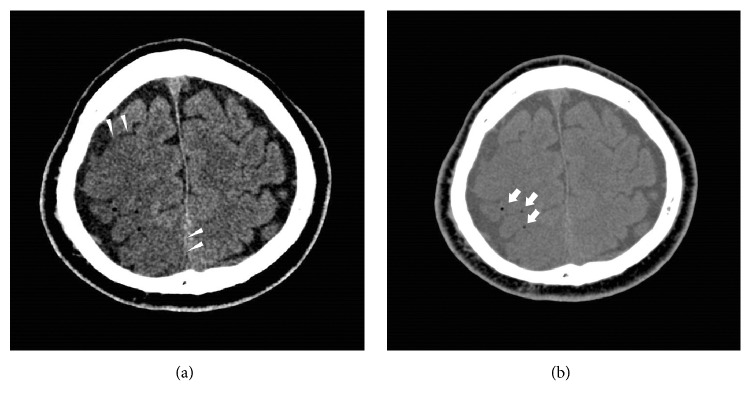
The same slice of head CT on the day of the onset of CAAE. The arrowheads show the obscureness of the corticomedullary junction. The arrows show the embolization of air bubbles. (a) Displayed with normal brain window settings (window width, 80 Hounsfield Units (HU); window level, 40 HU). (b) Displayed with mediastinal window settings (window width, 250 HU; window level, 40 HU).

At the second admission, flexible fiberoptic bronchoscopy was performed again under conscious sedation achieved by intravenous injection of 1.5 mg of midazolam. The patient was placed in the left lateral position and the cytological findings in the specimens obtained by curettage and instillation of saline into the right B^6^a were classified as class III. The patient had a little bleeding and coughing during the examination. After the examination, 0.2 mg of flumazenil was injected intravenously; however, the patient remained drowsy. Other vital signs were normal. When he became more alert three hours after the examination, he was found to have slurred speech, left hemiplegia, and left spatial neglect. The National Institute of Health Stroke Scale (NIHSS) score was 21. Emergency head CT revealed obscuring of the corticomedullary junction in the brain region supplied by the right middle cerebral artery (MCA) (Figure 2), and cerebral infarction was suspected. Reconstruction of the CT images using the mediastinal window settings revealed scattered air bubbles in the MCA region, based on which the patient was definitively diagnosed as having developed CAAE (Figure 2). Instead of hyperbaric oxygen (HBO_2_) therapy, which was not available at our hospital, we administered normobaric oxygen (NBO_2_) therapy (10 L/min of oxygen delivered via a nonrebreathing mask). The following day, the patient received fosphenytoin and levetiracetam for clonic convulsion extending from his left face to the upper arm. A repeat head CT showed disappearance of the air bubbles but emergence of cerebral edema, which improved over the following six days. While the patient was eventually able to walk using a four-legged cane, paresis of the left upper arm and left spatial neglect remained, and the Eastern Cooperative Oncology Group (ECOG) performance status was estimated to be 3. We therefore discontinued any further investigation or treatment of the lung cancer. Approximately four months after the second admission, the patient was transferred to a hospice near his hometown.

## 3. Discussion

This is the ninth reported case in the literature of CAAE developing after diagnostic flexible fiberoptic bronchoscopy. The profiles of the reported patients, the examination procedures undertaken, the treatments, and the outcomes are shown in Table 1 [4–10]. TBLB or TBNA was performed prior to the development of CAAE in all the previously reported cases. Our case was the first and only one to develop CAAE after bronchial curettage (strictly speaking, instillation of saline into the lesion of small volume in S^6^a may have induced CAAE by increasing the pressure of the peripheral airway). As curettage is a minimally invasive procedure, this case serves to underscore the importance for pulmonologists to be aware that any procedure that can cause bleeding could result in CAAE.

In patients undergoing CT-guided lung biopsy, location of the lesion in the lower lobe, occurrence of parenchymal hemorrhage, and use of a larger biopsy needle are all significant risk factors for the development of CAAE [3]. In contrast, the frequency of CAAE developing after diagnostic flexible fiberoptic bronchoscopy is too low to allow a statistically reasonable discussion of the risk factors for CAAE in patients undergoing bronchoscopy. However, we consider some shared factors between our case and some of the other eight cases as possible rick factors for CAAE occurring after bronchoscopy. In patients with chronic obstructive pulmonary disease (four cases, 44%), air from enlarged airspaces may easily flow into injured vessels. A cavity inside the mass (two cases, 22%) may also be a risk factor for the same reason. The diagnostic procedures were performed for lesions in the upper lobe in five (56%) cases and in S^6^ in three (33%) cases. No logical explanation for lesion location as a risk factor for CAAE can be provided based on this distribution; when the patient is in the sitting position, the lower pressure of the pulmonary veins in the upper lung field may lead to increased susceptibility to the development of CAAE, but this explanation would not be valid for patients in the supine position or lateral position, that is, during bronchoscopy. Before the onset of CAAE, the patient was laid in the left lateral position in two (22%) cases. No previous report has indicated the left lateral position as a risk factor for CAAE occurring during bronchoscopy or CT-guided lung biopsy, although air bubbles entering the left side of the heart may easily float up along the right side of the aortic wall into the right common carotid artery in this position, resulting in infarction of the right hemisphere. Our case fulfilled all of these four potential risk factors. Another factor to consider is bleeding during the procedure in two (22%) cases, because injury to the vessel wall may allow air to enter the vessel. Moreover, bleeding can induce coughs, which increase the intrabronchial pressure, leading to air embolism.

Definitive diagnosis of CAAE can be made when a CT image reveals air bubbles in the cerebral artery, but more subtle changes cannot be visualized on CT images. Therefore, some argue that visualization of air bubbles is not essential for the diagnosis of CAAE [11]. In one of the reported cases, no air bubbles could be visualized on the CT images, and the diagnosis was made by exclusion, that is, after confirming that there was no evidence of hypotension, hypoglycemia, or other causes of brain infarction [7]. Distribution of the ischemic lesions at the corticomedullary junction on brain magnetic resonance imaging (MRI) and refractory convulsions with little response to benzodiazepine administration are reported as findings that lend support to the diagnosis of CAAE [7, 12].

It is difficult to consider AAE in the differential diagnosis of complications occurring during bronchoscopy, because of its rarity. Moreover, we do not routinely perform CT to detect air bubbles after bronchoscopy. Therefore, asymptomatic AAE and self-limited air entry into the left atrium may have been missed. Although the frequency of AAE is likely lower in bronchoscopy than in CT-guided lung biopsy, the actual incidence of AAE in bronchoscopy may be higher than previously thought. For instance, some proportion of patients with cardiovascular events associated with bronchoscopy (71 cases, 0.068% in the previous survey [1]) may include those with subtle coronary AAE with normal coronary angiography, or cerebral AAE with normal brain CT. In our case, we detected CAAE in images reconstructed with mediastinal window settings, which helped in better visualization of the air bubbles. Figures 2(a) and 2(b) represent the same slice, but the air bubbles are more easily visualized in Figure 2(b). A recent paper in a radiological journal also pointed out the usefulness of window setting adjustments for visualizing air bubbles [13].

The recommended treatment for CAAE is HBO_2_ therapy. The US Navy manual for scuba diving recommends recompression to a pressure equivalent to that at a water depth of 60 feet (approximately 2.8 atmospheric pressures) immediately upon suspicion of AAE [14]. A definitive diagnosis is not essential for starting the therapy. The first rationale for using HBO_2_ therapy is that the hyperbaric environment reduces the volume of air bubbles, in accordance with Boyle's law (the gas volume is inversely proportional to the gas pressure). The second is that the low partial pressure of nitrogen in the blood makes it easy for the nitrogen gas in the bubbles to dissolve into the blood, in accordance with Fick's law (the diffusive flux is proportional to the concentration gradient). The third is that the high partial pressure of oxygen in the blood increases oxygen delivery to the damaged tissues [11]. When there is no access to a recompression chamber or HBO_2_ therapy is contraindicated, NBO_2_ therapy can be administered instead [5, 8, 11]. This satisfies the second and the third rationales for HBO_2_ therapy and has been shown to shorten the time taken for complete resolution of the air bubbles in dog studies [15].

In summary, we present the first case of CAAE developing as a complication of bronchial curettage performed during diagnostic flexible fiberoptic bronchoscopy. All pulmonologists should be aware of the possible occurrence of this complication after bronchoscopy, and should ensure prompt administration of supplemental oxygen, preferably HBO_2_ therapy, upon suspicion of AAE.

## Figures and Tables

**Table 1:** The profiles of the patients who developed CAAE after diagnostic flexible fiberoptic bronchoscopy.

Reference number	[4]	[5]	[6]	[6]	[7]	[8]	[9]	[10]	
Author and year	Shetty et al. 2001	Dhillon et al. 2004	Azzola et al. 2010	Azzola et al. 2010	Ragey et al. 2013	Evison et al. 2014	Goto et al. 2014	Tsuji et al. 2017	This case

Patient characteristics									
Age	60	55	60	68	70	84	69	51	77
Sex	Male	Male	Female	Female	Male	Female	Male	Male	Male
Underlying disease	N/A	COPD	N/A	N/A	COPD	N/A	N/A	COPD	COPD
Smoking history (pack-years)	N/A	N/A	N/A	N/A	30	N/A	0	30	55
Suspected disease	IPF	Lung cancer	Lung cancer	Lung cancer	Lung cancer	Lung cancer	Lung cancer	Tuberculosis	Lung cancer
Cavity in the mass	N/A	N/A	N/A	N/A	(−)	(−)	(−)	(+)	(+)

Bronchoscopy									
Examined lobe	N/A	RLL	LUL	RLL	LUL	RUL	LUL	RUL	RLL
Procedure	TBLB	TBNA, TBLB	TBNA, brush cytology, TBB	Brush cytology, TBNA	Brush cytology, TBNA	TBLB	TBNA, TBLB	Curettage, TBLB	Curettage
Positioning	N/A	Supine	N/A	N/A	N/A	N/A	Left lateral	Supine	Left lateral
Bleeding	N/A	50 mL	N/A	Minor	Little	N/A	Middle	Little	Little
Sedation	N/A	Meperidine, midazolam	Midazolam, hydrocodone	Hydrocodone, propofol	(−)	Midazolam, alfentanil	Pethidine	(−)	Midazolam

Diagnosis, treatment, and outcome of CAAE									
Lesion of infarction	Bilateral	Right frontal	Bilateral	Left hemisphere	Right hemisphere	Bilateral	Right postal	Left occipital	Right hemisphere
Air bubbles in the CT images	(+)	(+)	(+)	(+)	(−)	(+)	(+)	(+)	(+)
Oxygen delivery	HBO_2_	NBO_2_	HBO_2_	Intubation	HBO_2_	NBO_2_	HBO_2_	N/A	NBO_2_
Seizure	(+)	(+)	N/A	N/A	(+)	(+)	(−)	N/A	(+)
Outcome	Dead	Completely recovered	Dead	Dead	Completely recovered	Alive	Almost improved	Completely recovered	Partially improved

N/A, not available; COPD, chronic obstructive pulmonary disease; IPF, idiopathic pulmonary fibrosis; LUL, left upper lobe; TBLB, transbronchial lung biopsy; RUL, right upper lobe, RLL, right lower lobe; TBNA, transbronchial needle aspiration; TBB, transbronchial biopsy; HBO2, hyperbaric oxygen; NBO2, normobaric oxygen.
